# Do Lung Cancer Eligibility Criteria Align with Risk among Blacks and Hispanics?

**DOI:** 10.1371/journal.pone.0143789

**Published:** 2015-11-30

**Authors:** Kevin Fiscella, Paul Winters, Subrina Farah, Mechelle Sanders, Supriya G. Mohile

**Affiliations:** 1 Department of Family Medicine, University of Rochester Medical Center, Rochester, NY, United States of America; 2 Department of Public Health Sciences, University of Rochester Medical Center, Rochester, NY, United States of America; 3 Department of Medicine, Division of Oncology, University of Rochester Medical Center and the Wilmot Cancer Center, Rochester, NY, United States of America; National Health Research Institutes, TAIWAN

## Abstract

**Background:**

Black patients have higher lung cancer risk despite lower pack years of smoking. We assessed lung cancer risk by race, ethnicity, and sex among a nationally representative population eligible for lung cancer screening based on Medicare criteria.

**Methods:**

We used data from the National Health and Nutrition Examination Survey, 2007–2012 to assess lung cancer risk by sex, race and ethnicity among persons satisfying Medicare age and pack-year smoking eligibility criteria for lung cancer screening. We assessed Medicare eligibility based on age (55–77 years) and pack-years (≥30). We assessed 6-year lung cancer risk using a risk prediction model from Prostate, Lung, Colorectal and Ovarian Cancer Screening trial that was modified in 2012 (PLCO_m2012_). We compared the proportions of eligible persons by sex, race and ethnicity using Medicare criteria with a risk cut-point that was adjusted to achieve comparable total number of persons eligible for screening.

**Results:**

Among the 29.7 million persons aged 55–77 years who ever smoked, we found that 7.3 million (24.5%) were eligible for lung cancer screening under Medicare criteria. Among those eligible, Blacks had statistically significant higher (4.4%) and Hispanics lower lung cancer risk (1.2%) than non-Hispanic Whites (3.2%). At a cut-point of 2.12% risk for lung screening eligibility, the percentage of Blacks and Hispanics showed statistically significant changes. Blacks eligible rose by 48% and Hispanics eligible declined by 63%. Black men and Hispanic women were affected the most. There was little change in eligibility among Whites.

**Conclusion:**

Medicare eligibility criteria for lung cancer screening do not align with estimated risk for lung cancer among Blacks and Hispanics. Data are urgently needed to determine whether use of risk-based eligibility screening improves lung cancer outcomes among minority patients.

## Introduction

Lung cancer is the leading cause of cancer-mortality in the U.S. and represents an important health disparity.[1] Blacks have higher age-adjusted incidence of lung cancer and lower survival than non-Hispanic Whites.[1,2] Following a systematic review, including review of findings from the National Lung Screening Trial (NLST), [3] the United States Preventive Services Task Force (USPSTF) gave a grade B recommendation to annual lung cancer screening for smokers with low-dose computed tomography.[4]

This USPSTF recommendation has far-reaching implications for insurance coverage for lung cancer screening, medical liability related to failure to recommend screening for eligible patients, potential for overdiagnosis, harms and health care equity.[5–9] Under provisions of the Affordable Care Act (ACA), private insurers are required to cover the cost of preventive services that receive USPSTF grade B or higher recommendation.[10] In February 2015, the Center for Medicare & Medicaid services determined that Medicare would begin covering lung cancer screening with some stipulations.[11]

The USPSTF recommended eligibility for annual lung cancer screening according to criteria similar to enrollment criteria from the NLST.[3] Specifically, the USPSTF recommended use of a minimum of 30 pack-year smoking history among persons who have smoked within 15 years, including former smokers who have quit within this timeframe. Although the NLST used an age criteria of 55 to 74, the USPSTF extended the upper age limit to 80 years based on modeling of the risks and benefits.[9] Medicare adopted the same smoking criteria, but changed the age criteria to 55 to 77 years.

Age and pack-years of smoking criteria alone does not account for the higher lung cancer risk observed among Blacks compared with non-Hispanic Whites.[12] Use of these criteria alone may exclude some Blacks who may benefit from screening. The converse holds for Hispanics who have lower incidence of lung cancer than Whites.[2] These criteria may over-estimate risk for Hispanics.

Using nationally representative population data from the United States, we assessed the lung cancer risk among Whites, Blacks and Hispanics qualifying for lung cancer screening under Medicare eligibility criteria. We also compared numbers and percentage of ever smokers eligible under Medicare criteria with those based on a cut-point using a validated lung cancer risk prediction model.

## Materials and Methods

We assessed eligibility for lung cancer screening among participants in the 2007–2012 National Health and Nutrition Examination Survey (NHANES). We then assessed risk for lung cancer among these participants using the validated lung cancer risk prediction model, from the Prostate, Lung, Colorectal and Ovarian Cancer Screening trial, modified in 2012 (PLCO_m2012_).[13] Compared with NLST enrollment criteria, the PLCO_M2012_ criteria showed improved sensitivity and positive predictive value with no loss of specificity.[13] In a prospective evaluation, it out performs others risk prediction models.[14]

### Medicare eligibility criteria

We used Medicare eligibility criteria for lung cancer screening: 1) age (55–77 years); 2) smoking within 15 years; and 3) ≥30 pack-year smoking history. Exclusions included a history of lung cancer. In secondary analysis, we also excluded persons reporting poor health in response to a self-rated health question. This single item measure predicts both morbidity and mortality.[15,16]

### Assessment of lung cancer risk

We assessed risk for lung cancer among participants, ages 55–77 years, who had ever smoked regardless of pack years or duration of quitting. Screening never smokers has low yield.[17] We entered the following data regarding each participant into the risk scoring model: age (years); race/ethnicity (White, Black, Hispanic); educational attainment (< high school, high school [including post high school training], some college/associate degree, college graduate or greater); body mass index (kg/m^2^); prior history of cancer (yes/no); history of chronic obstructive pulmonary disease (yes/no); current smoking status (current/former); duration of smoking (years); smoking intensity (cigarettes/day); and smoking quit time (years quit). Family history of lung cancer was not collected in NHANES and was the only variable omitted from the risk factor model. We applied the PLCO_m2012_ lung cancer risk model based on the equations provided by the authors.[13]

### Comparison of eligibility based on Medicare and risk cut-point criteria

We compared eligibility based on Medicare criteria with that based on a cut-point for lung cancer risk that yielded the same total number of eligible persons. In sensitivity analyses, we examined the impact of alterative cut-offs and repeated the analyses after excluding persons who self-reported poor health.

### Weighting to generate national estimates

We used NHANES examination weights to account for oversampling and survey non-response and to derive national estimates for non-Hispanic Whites, non-Hispanic Blacks, and Hispanics. The weights were adjusted, according to analytic guidelines, for combining three cycle years.[18,19]

### Statistical Comparisons

We used the VARGEN procedure^15^ to estimate differences between weighted proportions.^16^ for participants eligible for screening under Medicare criteria and with PLCOm2012. We used SAS v 9.4 (Cary, NC) and SAS callable SUDAAN v 11.0.1 (Research Triangle Park, NC) statistical software.

## Results

Our derived estimated show that roughly half of the 60.7 million persons ages 55–77 (29.7 million) reported ever smoking. Among these ever smokers, half (15.1 million) reported smoking within the past 15 years. Among this group, 7.3 million (one in four of all ever smokers) were eligible for lung cancer screening based on Medicare ≥30 pack year eligibility criteria. Among the population of ever smokers, Blacks and Hispanics are more likely to currently smoke than Whites, but have lower pack-years of smoking. Among ever smokers ages 55–77 years, Whites have statistically significant (p <0.05) greater pack-years of smoking (29.5) than Blacks (21.4) or Hispanics (16.9). Blacks and Hispanics also have fewer years of education than Whites (Table 1).

**Table 1:** Risk Factors for Lung Cancer among Ever Smokers ages 55–77 years by Race and Ethnicity.

		Whites	Blacks	Hispanics
**Number of NHANES Participants**		1,304	676	582
**Population Estimate**		23,200,000	3,000,000	2,150,000
**Lung Cancer Risk Factor**	Category	%	%	%
**Age (years)**	55–59	33	30	38
	60–65	25	23	38
	66–69	26	28	23
	71–77	16	19	1
**Sex**	Male	57	53	65
	Female	43	47	35
**Education attainment**	< High School	31^†^	49^†^	62^†^
	High School	30^†^	22^†^	23^†^
	Some College	27^†^	22^†^	15^†^
	≥College Graduate	12^†^	7^†^	0^†^
**BMI (kg/m** ^**2**^ **)**	<18.5	2	6	0
	18.5–24.99	33	24	38
	25–29.99	33	31	31
	>30	32	39	31
**COPD**	Yes	10	3	0
	No	88	96	100
	Missing	2	1	0
**Smoking Status**	Current	54	62	69
	Former	46	38	31
**Duration of Smoking (yrs.)**	<20	1	0	0
	20–40	26	32	8
	41–60	70	66	92
	>60	3	2	0
**Smoking Intensity (cig/day)**	<10	8^†^	24^†^	8^†^
	10–20	49^†^	53^†^	61^†^
	>20	43^†^	23^†^	31^†^
**Duration of Quit Time (yrs.)**	0 (Current Smoker)	54^†^	62^†^	66^†^
	1–9	20^†^	17^†^	22^†^
	10–15	11^†^	20^†^	7^†^
	>15	15^†^	11^†^	5^†^

^†^ The proportions are different at 95% confidence limit between whites and blacks or Hispanics.

Among persons eligible for lung cancer screening under Medicare criteria, Black men had statistically significantly higher risk for lung cancer than White men while Hispanics had statistically significantly lower lung cancer risk than non-Hispanic Whites (Table 2). Sensitivity analyses that excluded those with poor health showed similar results.

**Table 2:** Lung Cancer Risk by Race, Ethnicity and Sex Among Ever Smokers Eligible for Lung Cancer Screening Under Medicare.

Sociodemographic characteristic	Estimated Number of Ever Smokers	Estimated Number of Eligible for screening**	Percent eligible for screening**	Median 6-year lung cancer risk*** (95% Confidence Interval)
**Total** *	29,740,000	7,250,000	24.4	3.20% (2.74, 3.65)
Men	17,100,000	4,530,000	26.5	3.06% (2.69, 3.72)
Women	12,640,000	2,720,000	21.5	3.26% (2.75, 3.86)
**Whites**	23,200,000	6,050,000	26.1	3.22% (2.74, 3.71)
Men	13,220,000	3,740,000	28.3	3.14% (2.68, 3.80)
Women	9,980,000	2,310,000	23.1	3.32% (2.74, 3.82)
**Blacks**	3,000,000	560,000	18.7	4.37% (3.86, 5.44)
Men	1,600,000	280,000^†^	17.5	4.39% (3.81, 5.21)
Women	1,400,000	280,000	20.0	4.32% (2.97, 5.85)
**Hispanics**	2,150,000	350,000^†^	16.3	1.18% (1.02, 1.59)
Men	1,390,000	260,000	18.7	1.34% (1.04, 1.89)
Women	760,000	90,000^†^	11.8	0.99% (0.47, 1.40)

^†^ Significantly different compared to whites at 95% confidence level.

*Includes persons of races other than black, white and Hispanic.

**Eligibility based on age (55–77), pack-years (≤30) and any smoking within 15 years.

***Based on PLCO_m2102_ model

We compared the proportion of ever smokers eligible for lung cancer screening by age, race, and ethnicity under Medicare criteria with lung cancer risk cut-point (2.12% 6-year risk) so that the size of the eligible population for the risk model population was comparable to the total size of the eligible population based on Medicare criteria. The risk-based eligibility criteria resulted in statistically significant changes in the percentage of minorities eligible for lung cancer screening (Table 3). Black men showed the largest percentage increase eligibility (12.4%) while Hispanic men showed the largest decrease (10.8%). Overall, the percentage of Black ever smokers increased by nearly half, from 18.7% to 27%. Conversely, the percentage of Hispanic ever smokers decreased by more than 60%, from 16.4% to 6.1%. There was no statistically significant change in eligibility among Whites. Data limitations, including racial specification beyond “other race” resulted in exclusion of Asians, American Indians, Alaskan natives, native Hawaiians/Pacific Islanders from these analyses.

**Table 3:** Differences by Race, Ethnicity and Sex in Percent of Ever Smokers Eligible between Medicare and Risk Criteria.

Sociodemographic Characteristics	Estimated number of persons eligible for screening based on risk cut-point*	Estimated percent of ever smokers eligible	Estimated change in percent of participants eligible based on risk cut-point relative to Medicare criteria**(95% CI)
**Whites**	6,050,000	26.1	0 (-2.9, 2.9)
Men	3,650,000	27.6	-0.7 (-4.7, 3.2)
Women	2,400,000	24.1	0.9 (-2.7, 4.5)
**Blacks**	830,000	27.7	8.8 (6.3, 11.3)†
Men	480,000	30.0	12.4 (9.2,15.7)†
Women	350,000	25.0	4.7 (0.3, 9.0)†
**Hispanics**	130,000	6.1	-10.1 (-13.4, -6.9)†
Men	110,000	7.9	-10.8 (-15.1, -6.5)†
Women	20,000	2.6	-8.9 (-13.2, -4.6)†

Numbers are in units of 10,000 persons. 95% CI = 95% Confidence Interval.

^†^ Significant at 95% confidence limit.

*cut-point = 2.12% six year risk

**Eligibility based on age (55–77), pack-years (30) and ever smokers. Eligibility is calculated on 2.12% six-year risk for lung cancer. This cut-point was derived by increasing the risk until the total number of persons eligible was comparable to eligible population based on age and pack years.

**Fig 1:**
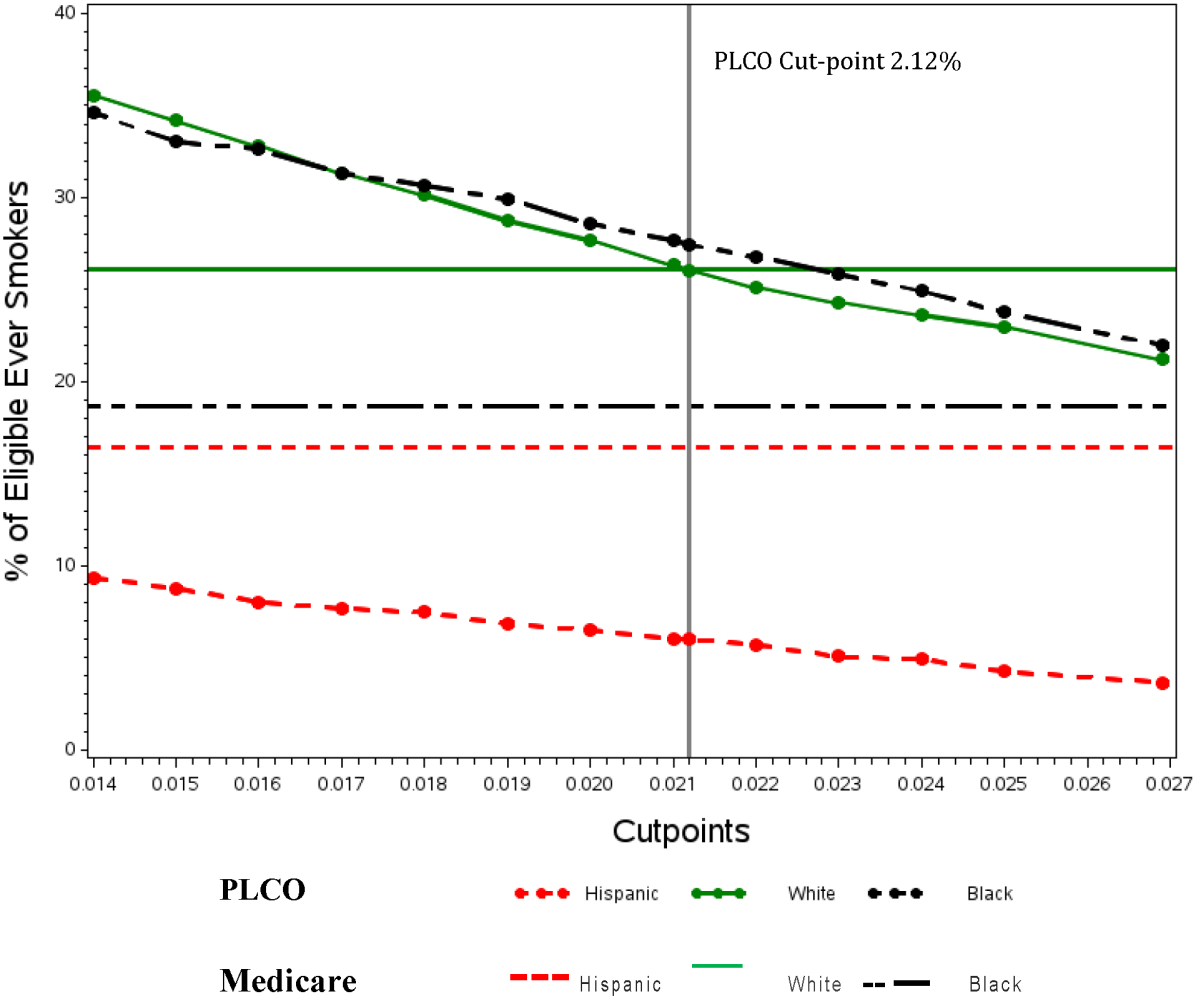
Comparison of Medicare lung cancer screening eligibility* with varying cut-points for PLCOm2012 by race/ethnicity. *Eligibility based on age (55–77), pack—year (30) and any smoking within 15 years.

We examined the effect of different cut points for the PLCO_m2012_. We varied cut-points from the 1.3455% used by Tammemagi *et al* [13] to twice this rate. The results are shown in the Fig 1. The horizontal lines show proportions of the population eligible by race and ethnicity using Medicare criteria. The sloping lines show these proportions based on changes in cut points. It is notable that the lines for non-Hispanic Whites happen to cross at the cut-point we used, 2.12%. In contrast, neither the lines for Blacks or for Hispanics cross. Use of a lower cut-point recently shown to optimize screening efficiency, i.e. 1.51%, [17] yielded similar findings though absolute numbers of Blacks, Whites, and Hispanics who are eligible for screening is higher when this lower threshold for eligibility is applied.

## Discussion

Current Medicare eligibility criteria for lung cancer screening do not align with lung cancer risk among Blacks and Hispanics. These criteria may exclude a substantial proportion of Blacks at higher lung cancer risk while including a high proportion of Hispanics at lower risk. Our findings show that use of risk model criteria based on the PLCO_m2102_ yield better alignment between eligibility for screening and known lung incidence for Blacks and Hispanics.[20,21] If lung cancer screening is shown to be effective when broadly adopted nationally, then improved alignment between screening eligibility and lung cancer risk could reduce disparities in lung cancer mortality. Conversely, current Medicare eligibility for screening could paradoxically increase racial disparities in lung cancer. Ma *et al* estimate that lung cancer screening could prevent more than 12,000 deaths annually based on an idealized scenario in which all eligible persons were screened and treated similarly as those enrolled in NSL.[22] If this estimate is correct, our findings suggest that these benefits would disproportionately accrue to non-Hispanic Whites due to better alignment of screening eligibility with predicted lung cancer risk. Based on disparities in screening and follow-up from existing cancer screening programs, [23] implementation of lung cancer screening based only on pack years could further accentuate these disparities resulting from misalignment between risk and screening eligibility.

Blacks, particularly males, have higher lung cancer incidence despite lower pack years.[12] The reasons for this paradox are not certain, but may reflect higher exposure to carcinogens, possibly related to inhalation, environments, and/or genetic metabolism.[24–26] These racial differences in pack-year risk have prompted the development of risk models such as the PLCO_m2012_ that explicitly capture these differences in lung cancer risk by race.[13] When the risk model is applied to the population of ever smokers, the proportion of Blacks eligible slightly exceeds that of Whites.

The PLCO_m2012_ also captures the lower lung cancer incidence of Hispanics. When this model is applied to Hispanics, relative eligibility drops further, particularly for women, reflecting in part the relatively younger age of Hispanic smokers Use of such risk models by clinicians could potentially yield better alignment of risk for lung cancer and screening eligibility for Blacks and Hispanics.

Findings from the NSLT show mortality benefit from lung cancer screening in the context of a large randomized trial that included academic medical centers and community-based radiology facilities.[3] Whether these findings will generalize more broadly is not certain.[27] Moreover, our findings do not address the question as to whether adoption of lung cancer risk models will translate into reductions in racial disparity in lung cancer mortality and/or potentially lower exposure to harms among those no longer eligible, particularly Hispanics. It is possible that the trade-offs in risks and harms for lung cancer screening might differ by race and/or ethnicity. Potential differences tumor biology, [28] morbidity, [29] affordability of diagnostic follow-up on screening, [30] and/or quality of care [31] could affect this trade-offs based on race/ethnicity.

Data are urgently needed to resolve the question of which eligibility criteria for lung cancer screening are most appropriate for minority patients. Medicare requires radiology imaging facilities to collect and submit data to a national registry for each LDCT lung cancer screening that is completed.[11] However, beyond a patient identifier and smoking history, there is no requirement for race and ethnicity data to be submitted for entry into the registry. The lung cancer screening registry, operated by the American College of Radiology, lists race and ethnicity as optional fields.[32] Although Medicare could conceivably match patient identifiers to their files to obtain race and ethnicity data, the reliability of these race/ethnicity data is lower for Hispanics than for Whites or Blacks.[33] Furthermore, there is no data field for patient educational level which is used by the PLCO_m2012_ to calculate risk. Absence of these data will hinder assessment of both predicted risk and outcomes among these groups, particularly Hispanics who have lower predicted lung cancer risk. Moreover, because data are only collected on those being screened, it will be difficult to determine based on registry data alone whether Medicare criteria are appropriate for racial or ethnic minority groups or not. Specifically, registry data by themselves will not directly inform benefits among those not satisfying the Medicare criteria. This will require analysis of a large prospective cohort among screened and unscreened persons using risk models applied to a sufficiently large number of Blacks and Hispanics. Last, development of alternative screening modalities for lung cancer, e.g. blood, might offer those who do not satisfy Medicare criteria for annual LDCT screening another screening option.[34]

Our findings are limited by absence of data regarding lung cancer family history among participants. Given higher incidence of lung cancer among Blacks and higher relative risk for family history among Blacks, [35] this limitation likely underestimates our estimate of lung cancer risk for African Americans. Our sample size limited the precision of estimates for subgroups, particularly Hispanic women where the number of ever smokers is smallest. Another limitation of our findings is our inability to generate risk estimates for minority groups such Asians, American Indians, and Pacific Islanders due to data limitations including small numbers. Previous studies have estimated that 8.6 million persons are eligible for lung cancer screening.[22] Our estimate of 7.3 million eligible for lung cancer screening is lower than previous estimates from the National Health Interview Survey because we accounted for smoking pack-years among eligible *former* smokers.

A potential obstacle to the use of risk models is low use by clinicians of computer or online risk calculators. A national survey showed few physicians reported using coronary artery risk tools.[36] The primary obstacle cited by these physicians was time.[36] A retrospective study that used electronic health record (EHR) lung cancer screening alerts based on age and pack years yielded high rates of lung cancer screening.[37] Conceivably, use of EHR alerts based on automated calculation of lung cancer risk could improve clinician use. Automated risk models for other conditions (e.g. coronary artery disease and heart failure re-admission) show promise.[38,39] However, limitations in EHR functionality and lack of relevant structured data within EHRs hinder widespread use of these automated risk calculators.

In conclusion, our findings suggest that Medicare eligibility criteria for LDCT lung cancer screening may not align with lung cancer risks for Blacks and Hispanics. They may exclude Blacks at higher lung cancer risk while including Hispanics at lower risk. This potential misalignment of risk could result in less benefit and/or greater harm to minorities than Whites from widespread implementation of lung cancer screening. Data are urgently needed regarding appropriate risk modeling for these minority groups to minimize unintended consequences of lung cancer screening on these groups.

## References

[1] Fox J, Richards C, Moolenaar R (2010) Racial/Ethnic disparities and geographic differences in lung cancer incidence-38 states and the District of Columbia, 1998–2006 Center for Disease Control and Prevention Morbidity and Mortality Weekly Report Atlanta, GA: Center for Disease Control and Prevention: 1433–1470.

[2] Kohler BA, Sherman RL, Howlader N, Jemal A, Ryerson AB, Henry KA, et al (2015) Annual report to the nation on the status of cancer, 1975–2011, featuring incidence of breast cancer subtypes by race/ethnicity, poverty, and state. Journal of the National Cancer Institute 107. 10.1093/jnci/djv048 PMC4603551 25825511

[3] National Lung Screening Trial Research Team, Aberle DR, Berg CD, Black WC, Church TR, Fagerstrom RM, et al (2011) The national lung screening trial: overview and study design. Radiology 258: 243–253. 10.1148/radiol.10091808 21045183 PMC3009383

[4] Moyer VA, US Preventive Services Task Force (2014) Screening for lung cancer: US preventive services task force recommendation statement. Annals of Internal Medicine 160: 9. 10.7326/M13-2771 24378917

[5] Aberle D, Abtin F, Brown K (2013) Computed tomography screening for lung cancer: has it finally arrived? Implications of the national lung screening trial. Journal of Clinical Oncology 31: 1002–1008. 10.1200/JCO.2012.43.3110 23401434 PMC3589698

[6] Field JK, Oudkerk M, Pedersen JH, Duffy SW (2013) Prospects for population screening and diagnosis of lung cancer. Lancet 382: 732–741. 10.1016/S0140-6736(13)61614-1 23972816

[7] Wender R, Fontham ET, Barrera E, Colditz GA, Church TR, Ettinger DS, et al (2013) American Cancer Society lung cancer screening guidelines. CA: a cancer journal for clinicians 63: 106–117. 10.3322/caac.21172 PMC3632634 23315954

[8] Patz E, Pinsky P, Gatsonis C, Sicks J, Kramer B, Tammemagi M, et al (2014) Overdiagnosis in low-dose computed tomography screening for lung cancer. JAMA Internal Medicine 174: 269–274. 10.1001/jamainternmed.2013.12738 24322569 PMC4040004

[9] de Koning HJ, Meza R, Plevritis SK, Ten Haaf K, Munshi VN, Jeon J, et al (2014) Benefits and harms of computed tomography lung cancer screening strategies: a comparative modeling study for the US preventive services task force. Annals of Internal Medicine 160: 311–320. 10.7326/M13-2316 24379002 PMC4116741

[10] Koh HK, Sebelius KG (2010) Promoting prevention through the affordable care act. New England Journal of Medicine 363: 1296–1299. 10.1056/NEJMp1008560 20879876

[11] Centers for Medicare & Medicaid Services Decision memo for screening for lung cancer with low dose computed tomography (LDCT)

[12] Haiman CA, Stram DO, Wilkens LR, Pike MC, Kolonel LN, Henderson BE, et al (2006) Ethnic and racial differences in the smoking-related risk of lung cancer. New England Journal of Medicine 354: 333–342. 16436765 10.1056/NEJMoa033250

[13] Tammemagi MC, Katki HA, Hocking WG, Church TR, Caporaso N, Kvale PA, et al (2013) Selection criteria for lung-cancer screening. New England Journal of Medicine 368: 728–736. 10.1056/NEJMoa1211776 23425165 PMC3929969

[14] Li K, Hüsing A, Sookthai D, Bergmann M, Boeing H, Becker N, et al (2015) Selecting high-risk individuals for lung cancer screening—a prospective evaluation of existing risk models and eligibility criteria in the German EPIC cohort. Cancer Prevention Research. 10.1158/1940-6207.CAPR-14-0424 26076698

[15] Latham K, Peek C (2012) Self-rated health and morbidity onset among late midlife US adults. The Journals of Gerontology Series B: Psychological Sciences and Social Sciences. 10.1093/geronb/gbs104 PMC3605944 23197340

[16] Idler EL, Benyamini Y (1997) Self-rated health and mortality: a review of twenty-seven community studies. Journal of Health & Social Behavior 38: 21–37. 9097506

[17] Tammemagi MC, Church TR, Hocking WG, Silvestri GA, Kvale PA, Riley TL, et al (2014) Evaluation of the lung cancer risks at which to screen ever- and never-smokers: screening rules applied to the PLCO and NLST cohorts. PLoS Med 11: e1001764 10.1371/journal.pmed.1001764 25460915 PMC4251899

[18] National Center for Health Statistics, Centers for Disease Control and Prevention (2013) National health and nutrition examination survey: analytic guidelines, 2011–2012.

[19] Johnson CL, Paulose-Ram R (2013) National health and nutrition examination survey: analytic guidelines, 1999–2010 25090154

[20] Siegel R, Naishadham D, Jemal A (2013) Cancer statistics, 2013. CA Cancer J Clin 63: 11–30. 10.3322/caac.21166 23335087

[21] Howe HL, Wu X, Ries LA, Cokkinides V, Ahmed F, Jemal A, et al (2006) Annual report to the nation on the status of cancer, 1975–2003, featuring cancer among U.S. Hispanic/Latino populations. Cancer 107: 1711–1742. 16958083 10.1002/cncr.22193

[22] Ma J, Ward EM, Smith R, Jemal A (2013) Annual number of lung cancer deaths potentially avertable by screening in the United States. Cancer 119: 1381–1385. 10.1002/cncr.27813 23440730

[23] Fiscella K, Humiston S, Hendren S, Winters P, Jean-Pierre P, Idris A, et al (2011) Eliminating disparities in cancer screening and follow-up of abnormal results: what will it take? Journal of Health Care for the Poor and Underserved 22: 83–100. 21317508 10.1353/hpu.2011.0023 PMC3647145

[24] Benowitz NL, Dains KM, Dempsey D, Wilson M, Jacob P (2011) Racial differences in the relationship between number of cigarettes smoked and nicotine and carcinogen exposure. Nicotine & Tobacco Research 13: 772–783. 21546441 10.1093/ntr/ntr072 PMC3168241

[25] St Helen G, Dempsey D, Wilson M, Jacob P, Benowitz NL (2012) Racial differences in the relationship between tobacco dependence and nicotine and carcinogen exposure. Addiction: 607–617. 10.1111/j.1360-0443.2012.04077.x 22971134 PMC3553231

[26] Berg JZ, Mason J, Boettcher AJ, Hatsukami DK, Murphy SE (2010) Nicotine metabolism in African Americans and European Americans: variation in glucuronidation by ethnicity and UGT2B10 haplotype. J Pharmacol Exp Ther 332: 202–209. 10.1124/jpet.109.159855 19786624 PMC2802474

[27] Bindman A (2015) JAMA Forum: lung cancer screening and evidence-based policy. JAMA 313: 17–18. 10.1001/jama.2014.16429 25562251

[28] Walsh KM, Gorlov IP, Hansen HM, Wu X, Spitz MR, Zhang H, et al (2013) Fine-mapping of the 5p15.33, 6p22.1-p21.31, and 15q25.1 regions identifies functional and histology-specific lung cancer susceptibility loci in African-Americans. Cancer Epidemiol Biomarkers Prev 22: 251–260. 10.1158/1055-9965.EPI-12-1007-T 23221128 PMC3565099

[29] Williams CD, Stechuchak KM, Zullig LL, Provenzale D, Kelley MJ (2013) Influence of comorbidity on racial differences in receipt of surgery among US veterans with early-stage non-small-cell lung cancer. J Clin Oncol 31: 475–481. 10.1200/JCO.2012.44.1170 23269988 PMC3731921

[30] Fox JB, Shaw FE (2015) Clinical preventive services coverage and the affordable care act. Am J Public Health 105: e7–e10. 10.2105/AJPH.2014.302289 PMC4265933 25393173

[31] Forrest LF, Adams J, Wareham H, Rubin G, White M (2013) Socioeconomic inequalities in lung cancer treatment: systematic review and meta-analysis. PLoS Med 10: e1001376 10.1371/journal.pmed.1001376 23393428 PMC3564770

[32] American College of Radiology Lung cancer screening registry.

[33] Zaslavsky AM, Ayanian JZ, Zaborski LB (2012) The validity of race and ethnicity in enrollment data for Medicare beneficiaries. Health Services Research 47: 1300 10.1111/j.1475-6773.2012.01411.x 22515953 PMC3349013

[34] Montani F, Marzi MJ, Dezi F, Dama E, Carletti RM, Bonizzi G, et al (2015) miR-test: a blood test for lung cancer early detection. Journal of the National Cancer Institute 107. 10.1093/jnci/djv063 25794889

[35] Coté ML, Kardia SL, Wenzlaff AS, Ruckdeschel JC, Schwartz AG (2005) Risk of lung cancer among white and black relatives of individuals with early-onset lung cancer. Jama 293: 3036–3042. 15972566 10.1001/jama.293.24.3036

[36] Shillinglaw B, Viera AJ, Edwards T, Simpson R, Sheridan SL (2012) Use of global coronary heart disease risk assessment in practice: a cross-sectional survey of a sample of U.S. physicians. BMC Health Serv Res 12: 20 10.1186/1472-6963-12-20 22273080 PMC3292915

[37] Federman DG, Kravetz JD, Lerz KA, Akg NK, Ruser C, Cain H, et al (2014) Implementation of an electronic clinical reminder to improve rates of lung cancer screening. American Journal of Medicine. 10.1016/j.amjmed.2014.04.010 24769298

[38] Persell SD, Lloyd-Jones DM, Friesema EM, Cooper AJ, Baker DW (2013) Electronic health record-based patient identification and individualized mailed outreach for primary cardiovascular disease prevention: a cluster randomized trial. J Gen Intern Med 28: 554–560. 10.1007/s11606-012-2268-1 23143672 PMC3599027

[39] Amarasingham R, Moore BJ, Tabak YP, Drazner MH, Clark CA, Zhang S, et al (2010) An automated model to identify heart failure patients at risk for 30-day readmission or death using electronic medical record data. Medical Care 48: 981–988. 20940649 10.1097/MLR.0b013e3181ef60d9

